# Cohesin in 3D: development, differentiation, and disease

**DOI:** 10.1101/gad.352671.125

**Published:** 2025-06-01

**Authors:** Maria Solé-Ferran, Ana Losada

**Affiliations:** Chromosome Dynamics Group, Molecular Oncology Programme, Spanish National Cancer Research Centre (CNIO), Madrid 28029, Spain

**Keywords:** embryogenesis, mouse models, cohesinopathies, Cornelia de Lange syndrome, chromosome organization, SMC

## Abstract

Cohesin contributes to genome spatial organization and sister chromatid cohesion. In this way, it not only supports accurate chromosome segregation and efficient DNA repair but also regulates gene expression. These functions are essential during embryonic development, the process that converts the fertilized egg into a complex organism with billions of specialized cells organized into tissues and organs. Here, we briefly summarize current knowledge on how cohesin and its associated factors perform chromatin looping and cohesion. We then review studies addressing their contribution to cell differentiation and embryonic development. Finally, we discuss cohesinopathies, human developmental syndromes caused by cohesin dysfunction.

## Abstract (precis)

In this review, Solé-Ferran and Losada discuss the mechanisms by which the cohesin complex facilitates chromatin looping and sister chromatid cohesion, processes essential for chromosomal segregation and spatial genome organization. They further highlight the importance of cohesin function during embryonic development and discuss the pathogenic mechanisms that drive developmental cohesinopathies.

Cohesin mediates sister chromatid cohesion and ensures accurate chromosome segregation during cell division, which is crucial for the rapid and precise proliferation of cells in a developing embryo (Guacci et al. 1997; Michaelis et al. 1997; Losada et al. 1998). The same complex translocates along DNA, generating progressively larger loops that are transiently stabilized at certain genomic positions (Rowley and Corces 2018). These chromatin loops help establish and maintain the spatial organization of the genome and facilitate the correct activation and repression of gene networks necessary for cell differentiation and tissue specification (Phillips-Cremins et al. 2013). Additionally, cohesin-mediated loop extrusion supports DNA damage repair, preserving genomic integrity during the extensive cellular divisions that characterize embryogenesis (Arnould et al. 2021). In view of its essential functions, it is not surprising that mutations in cohesin and its associated factors result in embryonic lethality or cause developmental disorders characterized by both intellectual and physical impairment. The most prevalent of these cohesinopathies is Cornelia de Lange syndrome (CdLS) (Kline et al. 2018). Here, we review the available information on the role of cohesin in cell differentiation and embryonic development, mostly obtained from mouse models, to better understand the consequences of its dysfunction in human disease.

## Cohesin and associated factors

**Figure 1.**
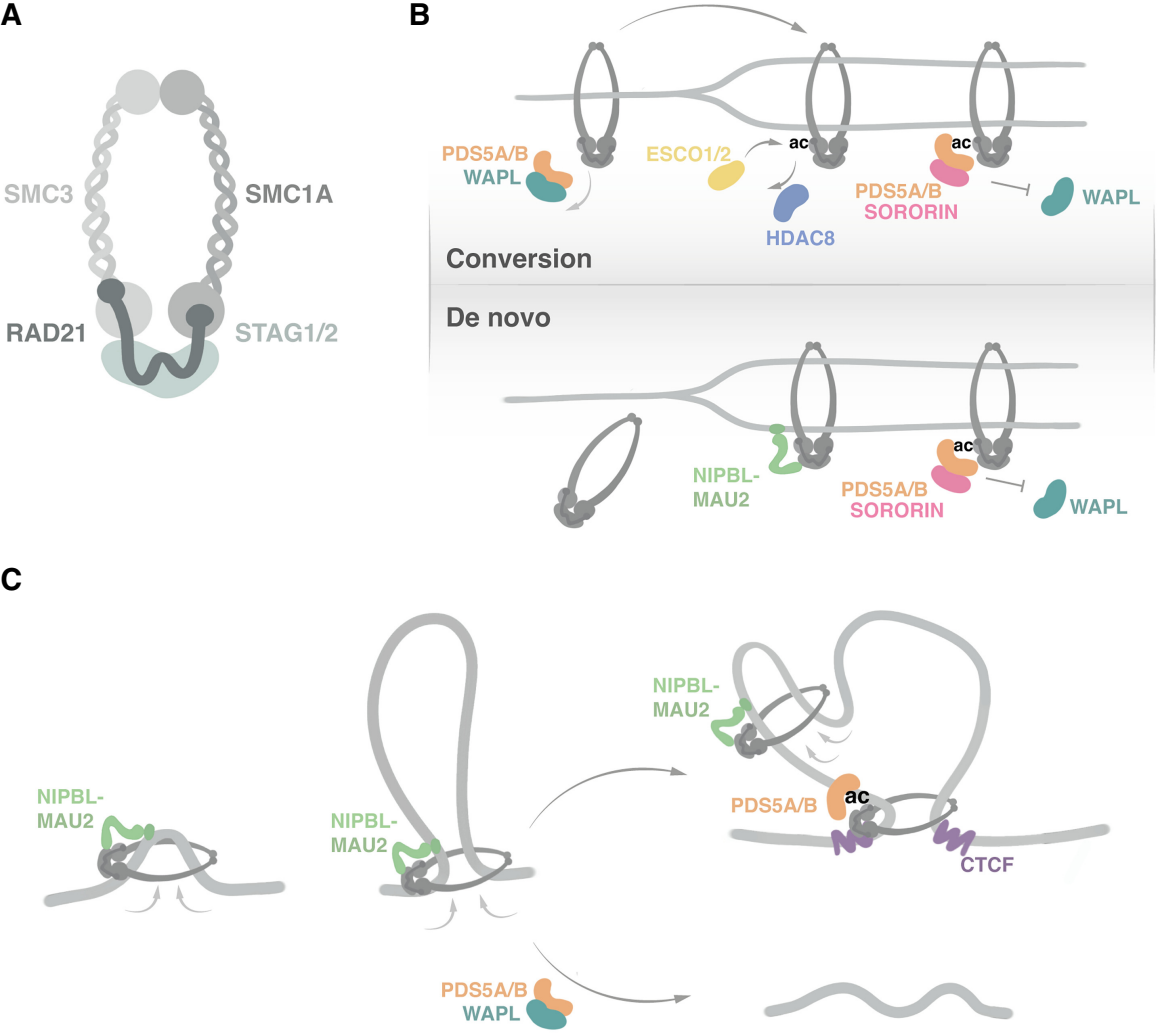
Cohesin composition and functions. (*A*) Schematic representation of the four-subunit cohesin complex. SMC1A and SMC3 form a V-shaped heterodimer with long coiled-coil arms folded over themselves and connected by a hinge domain. At the other end of the coiled coils, globular head domains are responsible for ATP binding and hydrolysis. RAD21 is a flexible, largely unstructured protein that bridges the SMC heads, effectively creating a ring structure that can entrap DNA. (*B*) The two pathways described for cohesion establishment during DNA replication. Both require the SMC3 acetylation and binding of SORORIN to PDS5A/B to stabilize cohesive complexes. Several replisome-associated proteins (not depicted) participate in this process. (*C*) Loop extrusion by cohesin entails binding and reeling in DNA to generate progressively larger loops, which requires binding to NIPBL–MAU2. Along the way, cohesin can either be released from chromatin by the action of WAPL–PDS5A/B, leading to dissolution of the loop, or become stalled at CTCF-bound sites, where it is acetylated and bound by PDS5A/B, which results in loop stabilization.

Cohesin is composed of structural maintenance of chromosomes 1A (SMC1A), SMC3, RAD21, and STAG, which exists in two variant forms: STAG1 and STAG2 (Fig. 1A; Yatskevich et al. 2019; Cuadrado and Losada 2020). Cohesin forms a ring that can entrap two pieces of DNA in *cis* (i.e., from the same chromatid) to form a loop or in *trans* to bring together the two sister chromatids after DNA replication. The association of cohesin with DNA is tightly regulated by several associated factors that make it dynamic for DNA looping but stable for cohesion. The heterodimer of NIPBL–MAU2 is essential for topological entrapment of DNA and to activate cohesin's ATPase (Murayama and Uhlmann 2014; Petela et al. 2018; Boardman et al. 2023). PDS5, with its two isoforms, PDS5A and PDS5B, competes with NIPBL for cohesin binding and facilitates the interaction of additional regulators with cohesin: WAPL, SORORIN, ESCO1, and HDAC8 (Kikuchi et al. 2016; Petela et al. 2018; Cuadrado et al. 2022; Nasmyth et al. 2023). WAPL promotes cohesin release from chromatin (Gandhi et al. 2006; Kueng et al. 2006; Tedeschi et al. 2013). Cohesin acetyltransferases (CoATs) ESCO1 and ESCO2 acetylate SMC3, a reaction reversed by histone deacetylase HDAC8 (Deardorff et al. 2012; Chan et al. 2013). Before DNA replication, only ESCO1 is present. CTCF, a zinc finger protein that binds DNA at thousands of sites within the genome, interacts with cohesin and also halts its progression, acting as a boundary element of chromatin loops (de Wit et al. 2015; Sanborn et al. 2015; Fudenberg et al. 2016; Li et al. 2020). After DNA replication, a fraction of cohesin encircling the two sister chromatids is acetylated by CoATs and bound by SORORIN, which stabilizes these cohesive complexes by preventing WAPL-mediated release until the cell enters mitosis (Nishiyama et al. 2010; Ladurner et al. 2016).

## Cohesin functions

Cohesin is a chromosome organizer with two major functions: sister chromatid cohesion and 3D genome architecture.

### Sister chromatid cohesion

During S phase, after passage of the replication fork, a fraction of cohesin complexes encircles the newly formed sister chromatids to hold them together until their segregation in mitosis (Nishiyama 2019). Cohesion establishment involves two distinct pathways (Fig. 1B; Xu et al. 2007; Srinivasan et al. 2020). In the “conversion” pathway, cohesin complexes already bound to unreplicated DNA are converted to cohesive cohesin in a mechanism that does not require NIPBL. Single-molecule imaging data suggest that this happens at sites of replication termination, where replication forks converge (Cameron et al. 2024). The second pathway involves de novo loading of cohesin onto replicated DNA and requires NIPBL, which is recruited to replicated DNA by PCNA (Zheng et al. 2018; Minamino et al. 2023; Psakhye et al. 2023). Cohesive cohesin complexes are acetylated and bound by SORORIN and therefore are resistant to unloading by WAPL (Nishiyama et al. 2010). The two CoATs can acetylate cohesin in S phase, but ESCO2 may be preferentially required for centromeric cohesion, at least in mice (Whelan et al. 2012; Kawasumi et al. 2017). Likewise, PDS5B has been shown to be important for centromeric cohesion (Carretero et al. 2013; Zhou et al. 2017).

At the onset of prophase, most cohesin complexes, cohesive or not, are removed from chromosome arms in a WAPL- and phosphorylation-dependent manner (Hauf et al. 2005; Gandhi et al. 2006; Kueng et al. 2006; Haarhuis et al. 2013). A small population of cohesin is protected from this “prophase” pathway by SGO1, which binds cohesin along chromosome arms and is then localized to centromeres (Liu et al. 2013; García-Nieto et al. 2023). SGO1–PP2A protects pericentromeric cohesin by dephosphorylating cohesin STAG subunits, SORORIN, and PDS5B (Nishiyama et al. 2013; Hellmuth and Stemmann 2024). These remaining cohesin complexes not only prevent premature separation of the sister chromatids but also facilitate proper attachment of the sister centromeres to the mitotic spindle (Sacristan et al. 2024). Once all chromosomes have achieved bipolar attachment, separase is activated and cleaves the RAD21 cohesin subunit, dissolving cohesion and allowing separation of the sister chromatids to opposite poles of the cell (Hauf et al. 2001; Shindo et al. 2022). In the absence of proper cohesion, aneuploidy is a likely outcome (Chunduri and Storchová 2019). Cohesin's role in holding sister chromatids together is also important for homologous recombination-mediated DNA repair in S and G2 phases, as the presence of a sister chromatid provides a template for accurate repair (Sjögren and Nasmyth 2001). Thus, cohesin ensures both the fidelity of chromosome segregation and the maintenance of genomic stability.

### Spatial genome organization

At the end of mitosis, cohesin reassociates with DNA to re-establish interphase chromosome organization (Abramo et al. 2019). Presumably, it does so through loop extrusion, a process in which cohesin translocates along the DNA, extruding progressively larger loops, until it is dissociated by WAPL or stalled by CTCF or some other obstacle (Fig. 1C; Davidson and Peters 2021; Dekker et al. 2023). An alternative proposal suggests that cohesin movement requires an extrinsic motor, most often an RNA polymerase (Guérin et al. 2024). Chromatin loops are transient structures (Gabriele et al. 2022; Mach et al. 2022). Regions of enriched contact density ∼1 Mb in size, known as topologically associating domains (TADs), emerge when averaging contacts from cell populations (Dixon et al. 2012; Flyamer et al. 2017; Bintu et al. 2018; Finn et al. 2019). In some cases, this organization ensures that genes are expressed in a context-specific manner, which is vital for cellular differentiation (Phillips-Cremins et al. 2013; Lupiáñez et al. 2015; Baudic et al. 2024; Chakraborty et al. 2025). However, there are also examples in which disruption of TAD boundaries does not result in obvious developmental defects (Despang et al. 2019; Williamson et al. 2019). DNA looping by cohesin brings distant regions of the genome into proximity, facilitating interactions between promoters and *cis*-regulatory elements, in particular those expanding >100 kb (Thiecke et al. 2020; Calderon et al. 2022; Kane et al. 2022; Rinzema et al. 2022; Robles-Rebollo et al. 2022). It also counteracts compartmentalization of chromatin into active and inactive domains and prevents stochastic coactivation of *cis*-linked genes (Schwarzer et al. 2017; Nuebler et al. 2018; Dong et al. 2024). TADs and most chromatin loops have cohesin and CTCF sites in convergent orientation at their borders (Rao et al. 2014; Sanborn et al. 2015; Nora et al. 2017). PDS5 proteins colocalize with cohesin complexes stalled at CTCF sites and recruit ESCO1 (Minamino et al. 2015). It has been proposed that SMC3 acetylation increases the binding affinity of cohesin for PDS5 and therefore disfavors binding of NIPBL, which prevents its translocation (Bastié et al. 2022; van Ruiten et al. 2022). The acetylation cycle involving ESCO1 and HDAC8 is an important determinant of loop length. In cell studies, PDS5A and PDS5B appear to be largely but not completely redundant (Wutz et al. 2017; Arruda et al. 2022; Cuadrado et al. 2022; Yu et al. 2022). PDS5A is dominant in human haploid HAP1 cells and mouse embryonic stem cells (mESCs), because its sole depletion leads to altered genome organization and derepression of Polycomb target genes, respectively (van Ruiten et al. 2022; Bsteh et al. 2023). It has also been shown that cohesin–STAG1 is preferentially found at CTCF sites and forms longer and more stable loops, whereas cohesin–STAG2 forms shorter and more dynamic loops, some of which are independent of CTCF (Kojic et al. 2018). This distinct behavior of the two variant cohesin complexes could be due to a different affinity for their regulators (Cuadrado et al. 2019, 2022; Wutz et al. 2020; Alonso-Gil et al. 2023). Cells lacking WAPL have very processive cohesin and form long loops (Haarhuis et al. 2017; Wutz et al. 2017). Modulation of cohesin dynamics by changes in WAPL abundance has been proposed as a key mechanism to establish different patterns of neural connectivity during brain development by regulating expression of clustered protocadherin (Pcdh) genes (Kiefer et al. 2023).

In addition to regulating transcription, loop extrusion is likely involved in recombination processes that generate T-cell receptor and antibody diversity (Seitan et al. 2011; Zhang et al. 2019; Hill et al. 2020). Active loop extrusion is also essential to resolve the two sister chromatids in G2 (Batty et al. 2023) and plays a role in DNA damage signaling, promoting formation of γH2AX-labeled DNA damage foci around DNA breaks, which in turn facilitates their repair (Arnould et al. 2021). Genome organization in TADs and compartments affects DNA replication dynamics and replication timing in somatic cells (Guillou et al. 2010; Emerson et al. 2022). Recent studies have shown that changes in DNA replication dynamics during cell fate transitions precede transcriptional changes and depend, at least in part, on genome organization (Nakatani et al. 2022; Ubieto-Capella et al. 2024). The time of emergence of replication timing programs in early mouse embryos and its connection with transcription are currently under debate (Halliwell et al. 2024; Nakatani et al. 2024; Takahashi et al. 2024; Xu et al. 2024).

## Cohesin and cell differentiation during embryonic development

**Figure 2.**
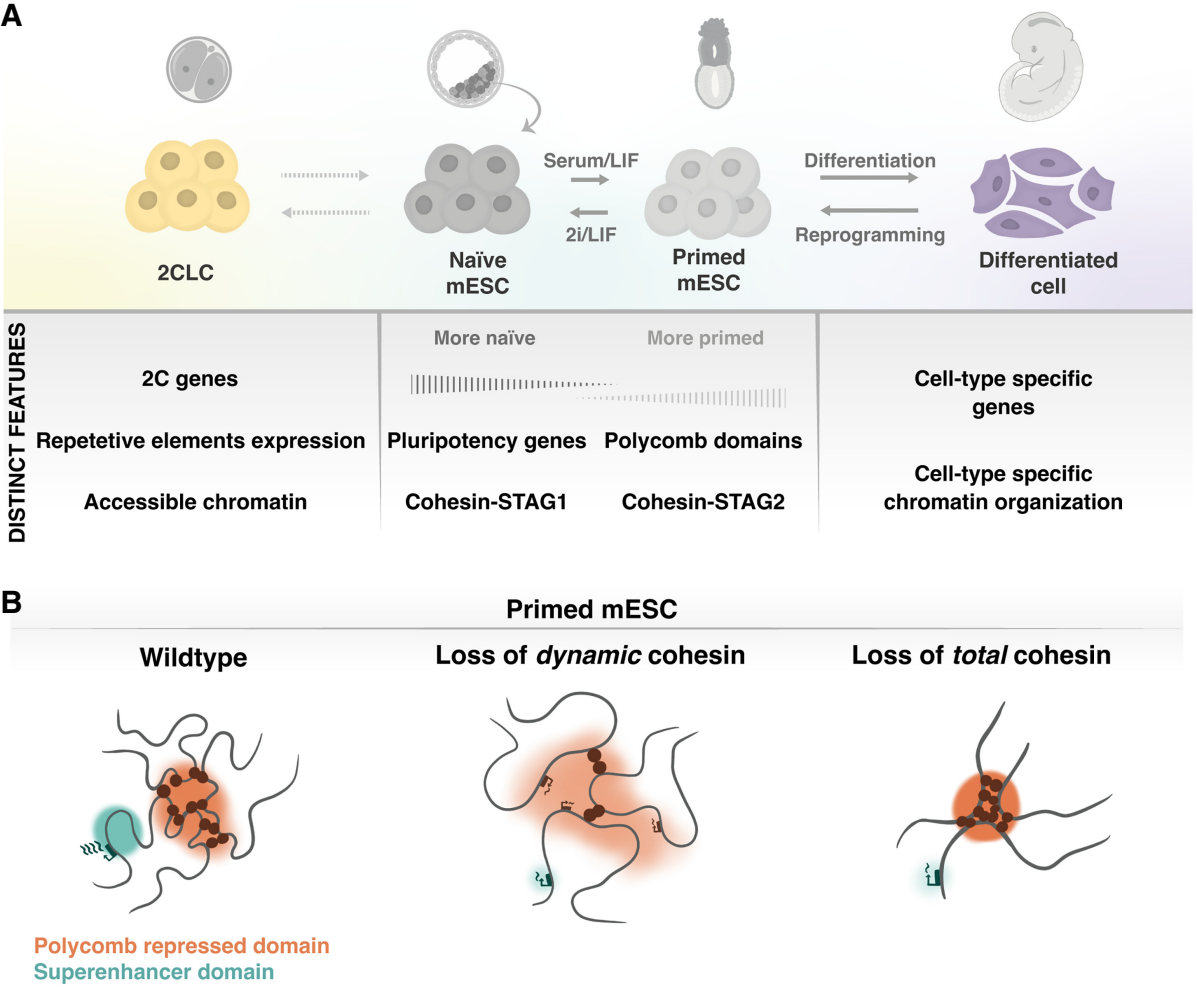
Cohesin and cellular differentiation in vitro. (*A*) Schematics of the cell states that can be recapitulated in cell culture mESCs obtained from the inner cell mass of the blastocyst. These cells can be cultured in medium containing a MEK inhibitor, a GSK3 inhibitor (2i), and leukemia inhibitory factor (LIF) to maintain self-renewal and pluripotency (naive state) or in serum/LIF to reach a “primed” pluripotent state in which Polycomb domains are established. During mESC culture, a transient totipotent-like state may spontaneously arise, known as the 2-cell-like cell (2CLC) state for its resemblance to the 2-cell blastomere. Additionally, mESCs can be differentiated to specific cell lineages, and, conversely, differentiated cells can be reprogrammed to acquire pluripotency. Distinct features of each of these cell states are indicated *below*. (*B*) Primed mESCs express pluripotency genes, controlled by superenhancer domains, and repress lineage-specifying genes in part through Polycomb complexes. Upon loss of dynamic cohesin (mostly cohesin–STAG2), superenhancer contacts and Polycomb domain contacts are reduced, leading to downregulation of pluripotency genes and upregulation of some lineage markers. Loss of total cohesin also leads to loss of superenhancer contacts and downregulation of pluripotency genes; however, it strengthens long-range interactions mediated by Polycomb.

Development begins with fertilization, leading to the formation of the totipotent zygote—a 1-cell embryo capable of giving rise to a complete organism. The embryo will then undergo several cleavage divisions, during which the nascent blastomeres become progressively more restricted in their developmental potential. Multiple differentiation events occur that must be tightly regulated to give rise to an individual with proper allocation and proportions of the different cell types. Some of these events can be recapitulated in vitro (Fig. 2A). Pluripotent mESCs, derived from the inner cell mass of blastocysts (E3.5), carry a very small fraction of 2-cell-like cells (2CLCs). These represent a transient population with characteristics of the 2-cell embryo and express totipotency genes such as *Zscan4*. Cohesin or CTCF knockdown (KD) in mESCs leads to upregulation of totipotency genes and increases the percentage of 2CLCs in the culture, possibly due to a more relaxed chromatin state (Olbrich et al. 2021; Zhu et al. 2021; Pezic et al. 2023). Specific culture conditions can maintain mESCs in a naive or primed pluripotent state, which resemble a preimplantation or a postimplantation embryo, respectively (Hackett and Surani 2014). The two states display differences in DNA methylation, in expression levels of pluripotency markers, and in the presence of H3K27me3 developmental genes bound by Polycomb complexes (Fig. 2A). The relative abundance of the two cohesin variants changes in the transition from naive to primed cells, with STAG1 levels going down and STAG2 levels going up, coinciding with the establishment of Polycomb domains (Fig. 2A; Cuadrado et al. 2019). These domains are formed by strong intrachromosome and interchromosome interactions that resemble compartments and keep developmental genes, such as *Hox* gene clusters, repressed (Schoenfelder et al. 2015; Du et al. 2020). Depletion of the dynamic cohesin by reducing STAG2 or WAPL disrupts these interactions, whereas total cohesin loss has the opposite effect, consistent with the role of loop extrusion in counteracting compartmentalization (Fig. 2B; Nuebler et al. 2018; Cuadrado et al. 2019; Rhodes et al. 2020; Kriz et al. 2021; Liu et al. 2021). At the same time, cohesin is important for pluripotency-specific gene expression, controlled by superenhancer regions (Liu et al. 2021). As a result, KD of cohesin or its regulators results in downregulation of pluripotency genes and upregulation of lineage-specifying genes (Kagey et al. 2010; Nitzsche et al. 2011; Apostolou et al. 2013; Cuadrado et al. 2019; Arruda et al. 2020).

Thus, perturbation of cohesin alters the pluripotent state of mESCs. Does it also affect differentiation? Some studies suggest that cohesin could be of particular importance when cells have to differentiate or respond to stimuli, as dynamic (more transient) cohesin-binding sites, chromatin contacts, and transcription change during differentiation (Bonev et al. 2017; Liu et al. 2021). Induction of mesoendodermal differentiation in SMC3 or NIPBL KD human ES cells results in reduced expression of several β-catenin-dependent mesoendodermal genes that rely on long-range chromatin contacts (Estarás et al. 2015). Likewise, acute loss of total cohesin in the context of a short differentiation protocol that converts mESCs into neural progenitor cells (NPCs) led to a reduction in transient interactions between some induced genes and their distal regulatory elements, negatively impacting their induction (Mahara et al. 2024). Dissolution of transient interactions between genes and Polycomb-occupied elements was also impaired. In another study, in vitro differentiation of NPCs to astrocytes after acute depletion of NIPBL resulted in mild dysregulation of a fraction of differentiation-specific genes (Hansen et al. 2024). When NIPBL-deficient mESCs were differentiated to gastruloids, embryonic organoids that recapitulate some aspects of postimplantation development, all cell lineages were produced, even if in slightly altered proportions (Hansen et al. 2024). However, the morphology of the gastruloids was clearly abnormal. These results suggest that transcriptional regulation through cohesin-mediated loop extrusion may not be critical for cell diversification during differentiation (which would mainly be driven by specific transcription factors) but may nevertheless contribute to appropriate cellular interaction or allocation in the different structures of the developing embryo. In support of this possibility, another study using gastruloids proposes that cohesin-mediated loop extrusion, in combination with CTCF directional insulation, drives sequential expression of genes along the *Hox* cluster (Rekaik et al. 2023). This sequential gene activation generates cell populations expressing distinct combinations of HOX factors along the anterior–posterior axis of the embryo that help specify the morphologies that establish the body plan. Shifts in *Hox* gene expression dynamics have been observed in *Nipbl*^+/−^ mouse embryos and after NIPBL KD in zebrafish pectoral fin buds (Kawauchi et al. 2009; Muto et al. 2014).

## Cohesin in mouse preimplantation development

**Figure 3.**
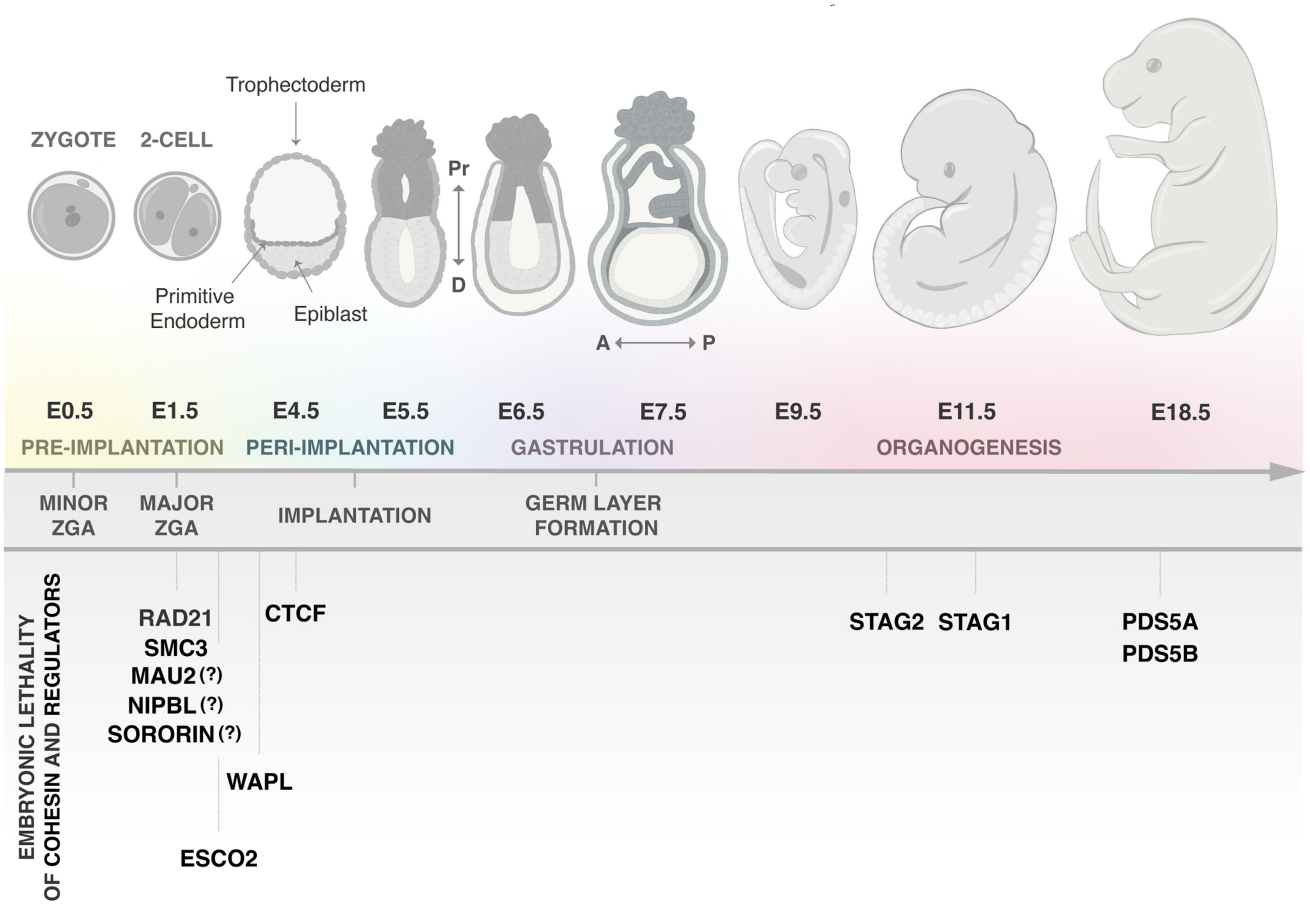
Mouse models of cohesin and regulators to study embryonic development. (*Top*) Time line of mouse development, indicated by embryonic (E) day after fertilization. Minor ZGA occurs in the zygote, and major ZGA occurs in the late 2-cell embryo. By E4.5, the embryo has three distinct cell types: the trophectoderm (TE) and the primitive endoderm (PrE), which will primarily give rise to extraembryonic tissues, and the epiblast (Epi), from which most of the embryonic tissues originate. The proximal–distal (Pr-D) axis is established during peri-implantation stages, whereas the anterior–posterior (A-P) axis is specified afterward. Following implantation, around E6.5, gastrulation will begin. This process involves dramatic cell migration and tissue rearrangement to establish the embryo's basic body plan and generation of the three primary germ layers: the ectoderm, mesoderm, and endoderm. This is the foundation for subsequent organogenesis, in which cells will be arranged into tissues and functional organs through cell–cell interactions and cell proliferation, migration, and fate determination events. (*Bottom*) The embryonic stage at which lethality is observed after genetic deletion of cohesin subunits and its regulators. For some of them, the exact time of lethality has not been determined (indicated by “?”) but is assumed to be early due to their essential role in cohesion. See also Table 1.

Epigenetic, transcriptional, and chromatin structural changes are tightly regulated during embryogenesis (Fig. 3, top). After fertilization, epigenetic remodeling, including global DNA demethylation, contributes to the establishment of totipotency in the zygote (Burton and Torres-Padilla 2014; Eckersley-Maslin et al. 2018). The initially quiescent zygotic genome becomes transcriptionally active in a process known as zygotic genome activation (ZGA), which occurs in two waves: minor and major ZGA (Abe et al. 2018a; Gassler et al. 2022). Chromatin loops and TADs can be observed already in zygotes but have weak boundaries that are strengthened over time (Du et al. 2017; Flyamer et al. 2017; Gassler et al. 2017; Ke et al. 2017). Compartmentalization also increases gradually. In the zygote, it is weaker in the maternal than in the paternal genome, but the difference fades out before the blastocyst stage (Du et al. 2017; Flyamer et al. 2017). After implantation, specification to the different germ layers and organogenesis are accompanied by the acquisition of specific transcriptional programs, epigenetic changes such as DNA methylation, and changes in chromatin architecture (Argelaguet et al. 2019; Nowotschin et al. 2019; Pijuan-Sala et al. 2019; Liu et al. 2023). During gastrulation, tight control of the spatial and temporal expression of *Hox* gene clusters is crucial for axial patterning, proper neural tube organization, or limb formation (Deschamps and Duboule 2017). The study of mouse embryonic development has been critical to understand embryogenesis, as well as to shed light on the underlying mechanisms driving human congenital disorders. Although there are notable differences between humans and mice, such as timing of ZGA, the morphology of the gastrulating embryo, or brain development, the fundamental processes that govern mammalian development are largely conserved.

Most cohesin-null mouse models are zygotic knockouts (KOs). Only two studies have depleted cohesin in oocytes to address the relevance of maternal contribution, but they provide important clues about the relevance of cohesin in very early embryos. Analysis of zygotes lacking RAD21 revealed that cohesin participates in the repair of DNA lesions acquired when the paternal genome is demethylated. In the absence of cohesin, the male pronucleus shows increased γH2AX-labeled foci and delayed entry into the first mitosis (Ladstätter and Tachibana-Konwalski 2016). DNA lesions also accumulate in SMC3-deficient zygotes, but these are only seen in G2, after DNA replication, and do not prevent entry into mitosis. In this case, chromosome segregation is severely impaired, with lagging chromosomes, elongated spindles, and generation of micronuclei in the first mitotic division, with subsequent arrest at the 2-cell stage. The differences between the phenotypes observed in RAD21- and SMC3-deficient zygotes likely depend on the amount of protein remaining after gene ablation in the oocytes (Yueh et al. 2021).

Chromatin loops and TADS were reduced in *Rad21* maternal–zygotic KO zygotes, but mitotic arrest prevented analyses in 2-cell embryos (Gassler et al. 2017). *Ctcf* KO embryos do not progress beyond the blastocyst stage and display transcriptional alterations in metabolic and protein homeostasis programs, most likely related to the presence of CTCF at the promoters of affected genes and not to a global defect in genome topology (Andreu et al. 2022). In zebrafish, cohesin is required for timely ZGA, but CTCF is not (Meier et al. 2018). In contrast, somatic cell nuclear transfer experiments showed that minor ZGA is facilitated by depletion of cohesin from donor cells (Zhang et al. 2020). Lack of cohesin possibly facilitates derepression of genes located in heterochromatin regions in the somatic cell, which is important for ZGA, whereas cohesin present in the receiving oocyte allows for normal development. The role of cohesin-mediated loop extrusion in (minor and major) ZGA remains to be clarified. The use of acute degradation (degron) approaches together with low-input technologies will help bypass the essential role of cohesin in chromosome segregation (Bondarieva and Tachibana 2024).

## Mouse models of cohesin and associated factors

Full depletion of cohesin subunits and most regulators in mice results in embryonic lethality at different stages of development, while heterozygous animals often display no obvious phenotypes (Table 1). Very early lethality is observed for deletion of unique core subunits (RAD21 and SMC3) and regulators (NIPBL, MAU2, WAPL, and SORORIN) (Fig. 3, bottom).

**Table 1.** Mouse models of cohesin subunits and regulators to study development

Protein	Null phenotype (zygotic)	Heterozygous phenotype	Null phenotype (tissue-specific)	References
RAD21	Early embryonic lethal	Reach adulthood, no morphological defects		Xu et al. 2010
			Cortex and hippocampal neurons (*Nex-Cre*), immature neurons	Calderon et al. 2022
SMC3	Early embryonic lethal	Reduced viability at P14, some morphological defects; brain alterations and increased anxiety-related behavior		White et al. 2013; Fujita et al. 2017
			Hematopoietic system *(Vav1-Cre)*, late embryonic lethality (>E13.5)	Wang et al. 2019
			Cardiac progenitors *(Nkx2.5-Cre)*, embryonic lethality before E14.5	Zhang et al. 2024
STAG1	Embryonic lethal (E11.5–17.5)	Increased incidence of spontaneous tumors		Remeseiro et al. 2012
STAG2	Embryonic lethal (E10.5)	X-linked, Stag2^+/−^ female embryos born at sub-Mendelian ratios		De Koninck et al. 2020
			Nervous system (*Nestin-Cre*), growth retardation; neurological defects, premature death	Cheng et al. 2022
NIPBL	Early embryonic lethal	High perinatal lethality, CdLS-Iike features (heart, craniofacial defects)		Kawauchi et al. 2009; Kean et al. 2022
MAU2	Early embryonic lethal	Reach adulthood, appear healthy		Smith et al. 2014
WAPL	Embryonic lethal (<E3.5)	Reach adulthood, appear healthy		Tedeschi et al. 2013; Kean et al. 2022
PDS5A	Late embryonic/perinatal lethality	Reach adulthood, appear healthy		Zhang et al. 2009; Carretero et al. 2013
PDS5B	Late embryonic/perinatal lethality	Reach adulthood, appear healthy		Zhang et al. 2007; Carretero et al. 2013
SORORIN	Early embryonic lethal	Reach adulthood, appear healthy		Ladurner et al. 2016
ESCO1	Born at sub-Mendelian ratios			Ajam et al. 2020
ESCO2	Lethal at 8-cell stage	Reach adulthood, appear healthy		Whelan et al. 2012
HDAC8	Perinatal lethality, skull instability	Partial lethality in females	Cranial neural crest cells (*Wnt1-Cre*), skull malformation	Haberland et al. 2009; Smith et al. 2014
CTCF	Embryonic lethal (E4.5)	Reach adulthood, appear healthy		Moore et al. 2012

### Mice deficient for genes encoding core cohesin subunits

*Rad21* heterozygous animals are viable and develop normally to adulthood without apparent morphological defects (Xu et al. 2010). They do have increased sensitivity to whole-body irradiation, most likely due to inefficient DNA repair. *Rad21* ablation in immature postmitotic neurons obtained from late embryos (E17.5/E18.5) disrupted cohesin/CTCF-mediated chromatin loops and decreased transcription of several neuronal genes, resulting in impaired neuronal maturation (Calderon et al. 2022). This study shows that the dependence on cohesin for proper gene expression directly correlates with the enhancer–promoter distance, consistent with the idea that cohesin is most needed to promote long-range contacts between *cis*-regulatory elements and their target genes (Kane et al. 2022).

*Smc3* heterozygous mice display reduced viability at P14, reduced body weight, and in some cases, a distinct craniofacial morphology (White et al. 2013). Using the same allele, another study reported no defects in heterozygous animals, whereas homozygous deletion in the hematopoietic system resulted in late embryonic lethality (Wang et al. 2019). Heart-specific deletion of both *Scm3* alleles with a *Nkx2.5-Cre* driver results in malformations in the outflow tract by E11.5 and death by E13.5 (Zhang et al. 2024). Altered transcription was identified in E12.5 cardiac tissues, but reduced proliferation possibly contributes to this phenotype as well. Because cohesin is essential for chromosome segregation, SMC3 depletion in cardiomyocytes must be incomplete. Another study focused on neural development found no alterations in brain anatomy or size in *Smc3* heterozygous mice (Fujita et al. 2017). However, these mice had greater dendritic complexity and more immature synapses in the cerebral cortex, as well as increased anxiety-related behavior. Transcriptome analyses detected enrichment of genes involved in the response to interferon γ, which is known to inhibit dendrite and synapse formation via the STAT1 transcription factor. Supporting the importance of this pathway, STAT1 KD partially rescued the synapse phenotype in cohesin-deficient neurons (Fujita et al. 2017). The relevance of cohesin for axon pruning had been previously reported in *Drosophila* postmitotic neurons, one of the first studies identifying a role for cohesin in nondividing cells (Pauli et al. 2008).

Unlike full elimination of RAD21 or SMC3, embryos lacking either STAG1 or STAG2 can survive well after gastrulation. This is a likely consequence of the overlapping roles of the two variant cohesin complexes in cohesion (van der Lelij et al. 2017). Lethality in *Stag1*-null mice starts around E11.5, with variable penetrance. No obvious morphological anomalies are observed in these embryos, and the cause of death is unclear. Heterozygous animals appear normal but have increased incidence of spontaneous tumors and a shorter life span than their wild-type littermates (Remeseiro et al. 2012).

*Stag2* is an X-linked gene, and S*tag2*-null male embryos show developmental delay by E9.5 and die by E10.5, possibly due to abnormal heart development. Specifically, embryos present anomalies in the right ventricle and the outflow tract, structures derived from the second heart field cardiac progenitors, resulting from a combination of transcriptional and proliferation defects (De Koninck et al. 2020). Heterozygous *Stag2* female embryos are present at the expected ratios by midgestation but are born at sub-Mendelian ratios. Those that reach adulthood appear as healthy as their wild-type littermates, though a more in-depth study of neurological abnormalities or aging was not pursued (De Koninck 2020). These animals might have benefited from skewed X inactivation toward the null allele. Deletion of *Stag2* in the nervous system using a *Nestin-Cre* driver leads to premature death (between 3 weeks and 4 months after birth), growth retardation, and neurological defects (Cheng et al. 2022). These defects are driven at least in part by impaired myelination of nerve fibers due to altered transcription of myelination-related genes. Hi-C analyses in oligodendrocytes revealed decreased numbers of promoter-anchored interactions in myelination genes, similar to the reduction observed in postmitotic neurons after deletion of *Rad2*1 and consistent with a predominant role of cohesin–STAG2 in enhancer–promoter contacts (Calderon et al. 2022).

### Mice deficient for genes encoding cohesin-associated factors

*Nipbl* heterozygous mice display a much stronger phenotype than those described above, and high perinatal mortality (Kawauchi et al. 2009). Mouse embryo fibroblasts from these animals express ∼70% of the *Nipbl* mRNA levels detected in wild-type cells, but this reduction appears to be sufficient to alter gene expression. Cellular NIPBL levels are usually lower than cohesin levels and are possibly rate-limiting for loop-extruding cohesin (Rhodes et al. 2017; Holzmann et al. 2019). CdLS-related features including impaired growth, craniofacial defects, and behavioral alterations are observed in the mutant mice. Heart anomalies including formation of the ventricular septum by E13.5, atrial septal defects, and reduced size of the right ventricle by E15.5–E17.5 can be observed in heterozygous embryos (Santos et al. 2016). These defects have been attributed to inefficient output of the second heart field progenitors starting at earlier stages, similar to what is observed in STAG2-deficient embryos by E10.5. Reduced cell proliferation, identified in *Stag2*-null embryos, is not expected in *Nipbl* haploinsufficient embryos, which may explain the earlier lethality of the former. Reduced expression of genes implicated in driving specific mesodermal lineages and failure to repress *Nanog* by the end of gastrulation likely contribute to the birth defects (Santos et al. 2016; Chea et al. 2024).

Brains of *Nipbl*^+/−^ embryos show a pattern of transcriptional dysregulation that partially overlaps with that of *Wapl*^+/−^ embryos. This result is consistent with the idea that a dynamic population of extruding cohesin complexes, which requires NIPBL and WAPL functions, facilitates enhancer–promoter contacts (Alonso-Gil and Losada 2023; de Wit and Nora 2023). Reduction of *Wapl* dosage partially rescues the gene expression changes of *Nipbl* mutant embryos and even their growth defect but not the perinatal lethality (Kean et al. 2022). Unlike *Nipbl*^+/−^ mice, *Wapl*^+/−^ mice are born at the expected frequencies, grow to adulthood, and appear healthy, whereas reducing *Wapl* dosage further with a single hypomorph allele results in death before weaning (Tedeschi et al. 2013; Kean et al. 2022).

*Mau2* heterozygous mice are also indistinguishable from wild-type littermates (Smith et al. 2014). The reasons for the different phenotypes resulting from reduced dosage of the two partners are unclear, because they stabilize each other (Parenti et al. 2020). It is possible that MAU2 is more abundant than NIPBL, and deletion of a single *Mau2* allele still provides sufficient MAU2 protein to stabilize wild-type levels of NIPBL. Inactivation of *Nipbl* or *Mau2* in neural crest cells causes severe malformations of the skull and jaws (Smith et al. 2014). Even though the *Wnt1-Cre* driver used in this study is active from E8.0 onward, expression of the neural crest marker *Sox10* was not affected at E9.5 and E10.5, and morphological differences between wild-type and mutant embryos were not observed until E13.5. Despite the essential nature of *Nipbl* and *Mau2*, cells continue to expand, most likely due to remaining levels of the corresponding proteins.

The *Cdca5* gene encoding SORORIN is essential for cohesion maintenance, but deletion of a single allele has no deleterious consequences for embryo development (Ladurner et al. 2016). For *Pds5A* and *Pds5B*, one study found late embryonic lethality for either gene KO (Carretero et al. 2013). However, other investigators reported that *Pds5A*-null and *Pds5B*-null mice die perinatally and show growth retardation, cleft palate, and skeletal defects (Zhang et al. 2007, 2009). Compound animals with a single *Pds5* allele, either *Pds5A* or *Pds5B*, survive to E11.5 and most likely die from heart anomalies (Zhang et al. 2009). Mice heterozygous for *Esco2* display no obvious phenotype. Chromosome segregation defects are observed at the 2-cell stage in full *Esco2* KO embryos, which do not survive past the 8-cell stage (Whelan et al. 2012). *Esco1*-null mice are born, though at sub-Mendelian ratios (16% instead of 25% expected from heterozygous mating) (Ajam et al. 2020). These results suggest that ESCO2 can compensate for the absence of ESCO1 but not the other way around. ESCO1's function in the regulation of loop-extruding cohesin may not be as critical for viability as ESCO2's function in cohesion establishment.

*Hdac8* is an X-linked gene. Hemizygous *HDAC8*-null mice display reduced size and die at weaning. In heterozygous females, partial lethality is observed, likely reflecting X-chromosome inactivation of the wild-type allele (Haberland et al. 2009). Lethality could be caused by skull instability, a phenotype that is also observed by conditional deletion of *Hdac8* in cranial neural crest cells. Transcriptional analyses in these cells uncovered aberrant expression of homeobox transcription factor genes such as *Otx2* and *Lhx1* in the absence of the deacetylase. These results indicate that, unlike cohesin acetylation, its deacetylation by HDAC8 is not essential for proliferation. The contribution of HDAC8 to gene expression is nevertheless important in some contexts, as shown for the cranial neural crest cells, but whether this contribution is mediated by cohesin remains to be studied.

## Human developmental syndromes caused by mutations in cohesin and its associated factors

Pathogenic variants in genes encoding cohesin subunits or its regulators cause a group of developmental syndromes collectively known as cohesinopathies. These include Cornelia de Lange syndrome (CdLS; the most prevalent), Roberts syndrome (RBS), and Warsaw breakage syndrome (WABS).

### Cornelia de Lange syndrome

**Figure 4.**
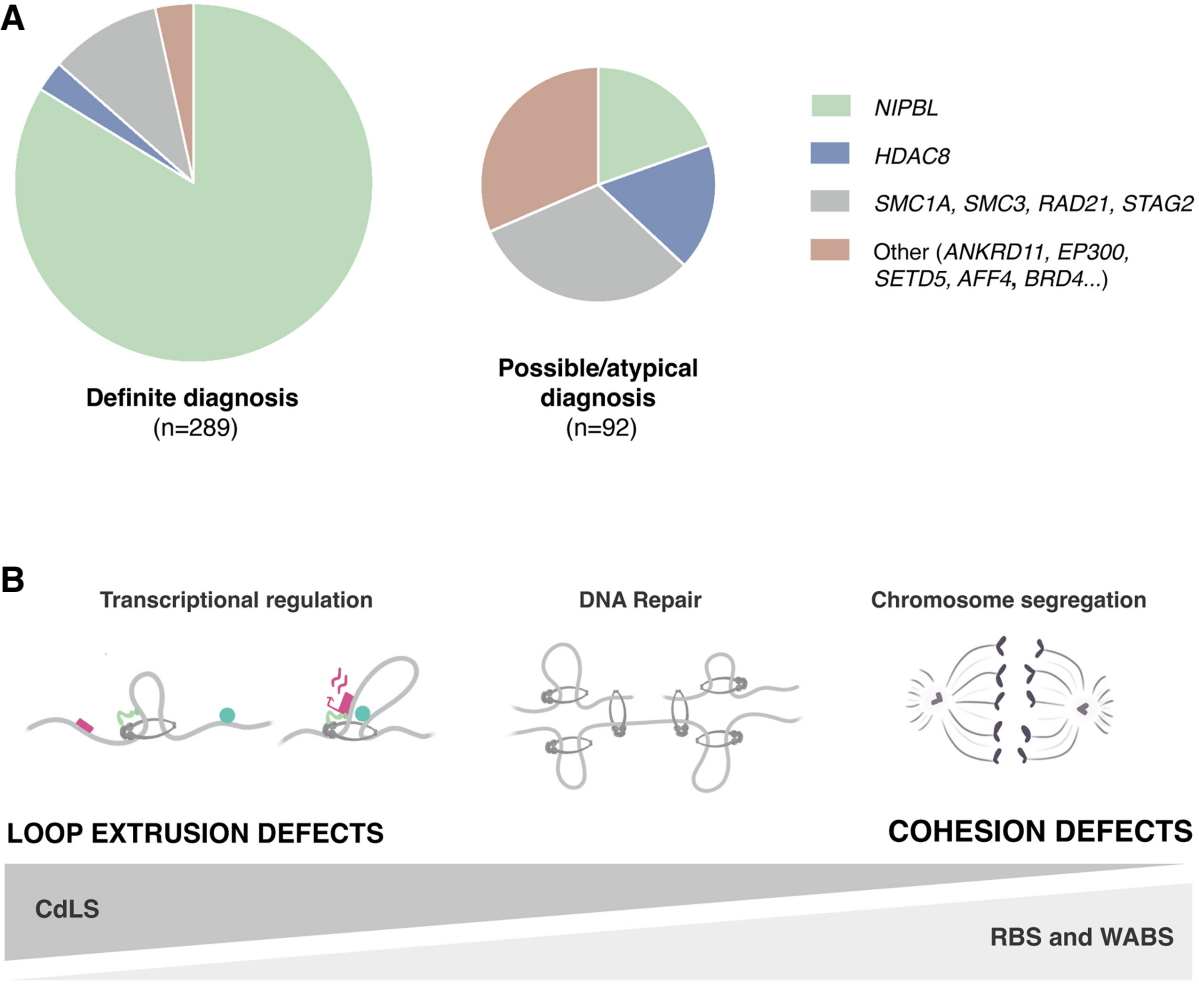
Cohesinopathies originate from mutations in genes encoding cohesin and its regulators. (*A*) Pie charts with mutation frequencies for the indicated genes in a cohort of 381 patients with a definite diagnosis of CdLS (*left* pie chart) or with less clear diagnosis (possible or atypical CdLS) (*right* pie chart), as reported by Kaur et al. (2023). *NIPBL* mutations are present in >80% of the former group. (*B*) Major functions of cohesin affected by mutations identified in cohesinopathy patients. While transcriptional alterations derived from loop extrusion defects are most important in CdLS, defects in DNA repair and chromosome segregation most likely underlie RBS and WABS.

CdLS affects 1:10,000–30,000 live births and is most frequently caused by loss-of-function heterozygous mutations in *NIPBL* (Kline et al. 2018; Kaur et al. 2023). It is a multisystemic disorder characterized by distinct craniofacial features, growth retardation both before and after birth, intellectual disability, behavioral issues, and limb malformations. Mutations in *SMC1A*, *SMC3*, *RAD21*, and *HDAC8* have been identified in a small fraction of patients (Fig. 4A). A short deletion in the N-terminal region of *MAU2* has also been reported (Parenti et al. 2020). Individuals with mutations in *STAG1* and *STAG2* present phenotypes partially overlapping with CdLS (Yuan et al. 2019).

The finding of mutations in transcriptional regulators (*ANKRD11*, *AFF4*, *ARID1B*, *BRD4*, *EP300*, and *SETD5*, among others) in patients with CdLS-like features adds to the mounting evidence that supports the idea that CdLS is caused by defects in transcription (Fig. 4A; Izumi et al. 2015; Parenti and Kaiser 2021). Analyses in cells from CdLS patients and mouse models of *NIPBL* haploinsufficiency reveal altered gene expression but not cohesion defects (Liu et al. 2009; Remeseiro et al. 2013; Boudaoud et al. 2017; Newkirk et al. 2017; Garcia et al. 2021). Transcriptional profiling of cortical neurons from CdLS patients showed dysregulation of many genes related to neuronal functions, which could be partially recapitulated by elimination of cohesin in postmitotic cortical mouse neurons (Weiss et al. 2021). Induced pluripotent stem cells (iPSCs) reprogrammed from the leukocytes of a CdLS patient carrying a missense *NIPBL* mutation were more difficult to differentiate to the hepatic lineage than mutation-corrected cells (Foglia et al. 2024). Intriguingly, chromatin immunoprecipitation analyses in the iPSCs appeared to indicate increased cohesin on chromatin genome-wide. In contrast, another study using fibroblasts from CdLS patients reported reduced NIPBL occupancy and redistribution of cohesin along the genome, with decreased presence at CTCF positions and increased presence at NIPBL sites, suggesting impaired loop extrusion (Garcia et al. 2021). Reconstitution of loop extrusion activity with NIPBL proteins carrying some CdLS mutations has shown that at least some of these mutations result in decreased extrusion efficiency (Panarotto et al. 2022). Additional mechanisms could be at play to explain the consequences of partial reduction of functional NIPBL on gene expression. Recent results comparing immortalized skin fibroblasts from a *NIPBL* mutant CdLS patient and an *AFF4* mutant CHOPS patient (a CdLS-like syndrome) revealed no major differences in TADs and compartments (Sakata et al. 2025). However, a reduction in cohesin, NIPBL, BRD4, and acetylated H3K27 at enhancers that correlates with weaker enhancer–promoter looping and reduced gene expression was observed in both patient mutant cells compared with those of a healthy individual. Developmental genes were among the most affected. Whether impaired loop extrusion, impaired enhancer activation, or both contributes to gene downregulation is unclear. Also unclear is how increased levels of a core component of the superelongation complex (SEC; the A*FF4* mutation enhances protein stability) has effects that are similar to a reduction of NIPBL. It is possible that the SEC affects BRD4 binding at these developmental enhancers, and this in turn negatively affects NIPBL recruitment. In fact, BRD4 has been shown to bind and stabilize NIPBL at enhancers required to promote neural crest progenitor cell differentiation (Linares-Saldana et al. 2021).

According to single-cell RNA sequencing, NIPBL heterozygous mouse embryos have all the cell populations present in their wild-type littermates at the time of gastrulation, but changes in the number of cells in the mesodermal lineage are observed (Chea et al. 2024). Whether this is the consequence of transcriptional alterations alone is unclear. Subtle changes in proliferation may affect development of complex organs such as the heart or brain, which requires a careful coordination of the maturation of their distinct structures. In developing hearts of STAG2-deficient embryos, both proliferation and transcription defects were identified (De Koninck et al. 2020). Additional pathological mechanisms may be related to DNA damage and senescence. An increased number of 53BP1 foci was observed in two lymphoblastoid cell lines derived from CdLS patients harboring mutations in *NIPBL* in the absence of any external stress (Olley et al. 2021). Recovery after irradiation is sometimes affected in fibroblasts from CdLS patients (Remeseiro et al. 2013; Di Nardo et al. 2024). Persistent DNA damage was detected in the placentas of NIPBL- and HDAC8-deficient mice, leading to enhanced senescence in trophoblast stem cells and increased secretion of inflammatory cytokines (Singh et al. 2020). Providing wild-type placentas to mutant embryos partially rescued ossification and growth defects, suggesting that at least part of these CdLS-related defects may derive from placental anomalies. Cohesin has been proposed to participate in DNA repair and DNA damage signaling in several ways, including loop extrusion-mediated spreading of γH2AX and regulation of homology searches (Arnould et al. 2021; Piazza et al. 2021).

A recent study showed that in female mice that carry one wild-type allele and one *Stag2* mutant allele deficient in the interaction with CTCF, the latter contributes to most tissues at the expected frequencies except for lymphocytes (Buenaventura et al. 2024). In contrast, in homozygous mutant females and hemizygous males (that is, in the absence of wild-type STAG2), functional lymphocytes are properly generated. These results suggest (1) that the ability of cohesin–STAG2 to arrest at CTCF sites is not essential for formation of most tissues, including the lymphocyte compartment, and (2) that the failure of *Stag2* mutant clones to contribute to this compartment, which is only observed in the presence of the wild-type allele, could be driven by some sort of X-linked cell competition. A similar process appears to take place in human cells and affect cohesinopathy female patients with mutations in *STAG2* or *HDAC8*, as they show skewed X inactivation of the mutant alleles in their blood (Renault et al. 2011; Kaiser et al. 2014)

Taken all together, it is likely that a combination of minor alterations in transcription—but also in DNA repair or even in proliferation—disturbs embryo development and results in the many different malformations reported for CdLS patients (Fig. 4B).

### Roberts syndrome and Warsaw breakage syndrome

Roberts syndrome is characterized by limb and craniofacial abnormalities, as well as growth and intellectual impairment. It is caused by homozygous mutations in *ESCO2* (Vega et al. 2005). This CoAT is present in cells in S/G2 phase and has a major role in cohesion establishment, though its loss can be partly compensated by ESCO1 (Alomer et al. 2017). Premature centromere separation is observed in metaphase chromosomes of RBS patients. *Esco2* deficiency is lethal in murine embryos, perhaps because the acrocentric nature of mouse chromosomes makes them more sensitive to centromeric cohesion loss (Whelan et al. 2012). Conditional deletion of *Esco2* in the developing limbs of murine embryos results in perinatal lethality, with mutant pups showing a severe limb reduction, disorganized chondrocytes, and a poorly developed chondroid matrix (Strasser et al. 2024). Transcriptome analyses in E9.5 embryos before detection of abnormal limb morphology and hemorrhage revealed upregulation of genes related to p53 signaling. This could be driven by DNA damage induced by reactive oxygen species (ROS) production, a phenotype observed in *Eco1* mutations in yeast and human RBS cell lines (Mfarej and Skibbens 2022). In utero treatment with a p53 inhibitor rescued the hemorrhage, whereas defects in chondrogenesis persisted, suggesting a different cause, perhaps related to altered gene regulation (Strasser et al. 2024). To date, however, there are no strong data to support a role of ESCO2 in cohesin-dependent or cohesin-independent regulation of genome organization and transcription.

Like RBS, metaphase chromosomes in cells from Warsaw breakage syndrome patients display cohesion defects including a lack of a primary constriction (van der Lelij et al. 2010). This disorder is caused by biallelic mutations in the gene coding for DDX11, a DNA helicase involved not only in cohesion establishment through the conversion pathway mentioned above, but also in DNA damage tolerance (Abe et al. 2018b). Mutant alleles restore sister chromatid cohesion when overexpressed in WABS patient cells, showing that they are hypomorphic mutations (van Schie et al. 2020). Mouse embryos lacking DDX11 display placental defects and do not survive past E10.5 (Inoue et al. 2007; Cota and García-García 2012). Delayed growth and widespread apoptosis are observed in mutant embryos already at E7.5, and cellularity is very sparse at late stages despite the presence of a body axis. Defects in chromosome segregation and genome instability likely underlie the pathophysiology of RBS and WABS (Fig. 4B). Whether these defects in turn affect differentiation programs is unclear.

## Outlook

Despite the major advances in our understanding of how cohesin works at the molecular level, many questions remain open. These include whether different mechanisms are at play in cohesion and chromatin loop formation, how the loop extrusion process observed in vitro recapitulates what happens in vivo in the crowded environment of the nucleus, and how chromatin-bound complexes interact with cohesin and affect its genome-folding dynamics both globally and locally (Guo et al. 2022; Barth et al. 2025; Tjalsma et al. 2025; Uhlmann 2025). In turn, how this dynamic folding regulates gene expression, DNA replication, or DNA repair is still unclear, and novel cohesin interactors and even novel functions of the complex have emerged in recent studies (Mayayo-Peralta et al. 2023; Singh et al. 2023; van Schie et al. 2023; Fedkenheuer et al. 2025; Nguyen et al. 2025; Tang et al. 2025). Uncovering the role of cohesin in development and differentiation has remained challenging due to its essential role in chromosome segregation. Identification of cohesin mutations abolishing loop extrusion while allowing cohesion establishment and vice versa would help in understanding the distinct contributions of these two functions of cohesin during embryonic development (Nagasaka et al. 2023). The use of degron-tagged versions of cohesin and/or its regulators to achieve acute protein ablation at selected times during development or in the context of stem cell-derived embryo models could be a useful approach for the detailed exploration of cohesin's role in embryogenesis (de Wit and Nora 2023; Shahbazi and Pasque 2024). The combination of advanced imaging technologies, low-input/single-cell genomic approaches, and spatial transcriptomics will permit characterization of these in vitro and in vivo experimental models with unprecedented detail. To better understand the pathological consequences of cohesin mutations, patient-derived human-induced pluripotent stem cells (hiPSCs) are already being used in differentiation assays to uncover their effects on cell lineage commitment through the integration of chromatin contact maps, transcriptomics, and epigenomic landscape profiling (Mills et al. 2018; Foglia et al. 2024). Systematic analyses of DNA repair and chromosome segregation, as well as biochemical studies of the consequences of the mutation in protein folding, protein interactions, or chromatin association dynamics, should also be performed to decipher the molecular mechanisms driving disease. This knowledge may improve clinical management of the patients and even shed light on future therapeutic strategies.
